# Division of labor, specialization and diversity in the ancient Roman cities: A quantitative approach to Latin epigraphy

**DOI:** 10.1371/journal.pone.0269869

**Published:** 2022-06-16

**Authors:** Vojtěch Kaše, Petra Heřmánková, Adéla Sobotková

**Affiliations:** 1 Department of Philosophy, University of West Bohemia, Plzeň, Czech Republic; 2 Department of History and Classical Studies, Aarhus University, Aarhus C, Denmark; University at Buffalo - The State University of New York, UNITED STATES

## Abstract

Recent empirical studies on the division of labor in modern cities indicate a complex web of relationships between sectoral specialization of cities and their productivity on one hand and sectoral diversification and resilience on the other. Emerging scholarly consensus suggests that ancient urbanism has more in common with modern urban development than previously thought. We explore whether modern trends in urban division of labor apply to the cities of the Western Roman Empire from the first century BCE to the fourth century CE. We analyze occupational data extracted from a large body of Latin epigraphic evidence by computer-assisted text-mining, subsequently mapped onto a dataset of ancient Roman cities. We detect a higher frequency of occupation terms on inscriptions from cities led by Rome than from rural areas and identify an accumulation of tertiary sector occupations in large cities. The temporal dimension of epigraphic data allows us to study aspects of the division of labor diachronically and to detect trends in the data in a four centuries-long period of Roman imperial history. Our analyses reveal an overall decrease in the frequency of occupational terms between the first half and second half of the third century CE; the maximum frequency of occupational terms shifts over time from large cities to medium and small towns, and finally, rural areas. Our results regarding the specialization and diversity of cities and their respective impact on productivity and resilience remain inconclusive, possibly as a result of the socio-economic bias of Latin inscriptions and insufficient representativeness of the data. Yet, we believe that our formalized approach to the research problem opens up new avenues for research, both in respect to the economic history of the Roman Empire and to the current trends in the science of cities.

## Introduction

Our current ability to evaluate socioeconomic development of past societies depends on a combination of three ingredients: access to relevant data, adoption of appropriate methods, and employment of sufficiently robust theoretical framework. In this article, we offer a novel mixture of these ingredients in an attempt to assess several aspects of the socioeconomic development of the Roman Empire. We employ ancient epigraphic evidence, methods of computational text analysis and Monte Carlo simulations and theoretical insights on scaling of cities to answer questions such as: What is the structure of the Roman labor force across individual cities and the Empire? How does division of labor evolve over time? How does occupational specialization and diversification differ from city to city?

The human experience with urbanism covers approximately 7,000 years [1], during which cities emerged independently in many different parts of the world [2]. Perhaps the most defining feature of cities is how they intensify social interactions, which led some scholars to characterize them as *social reactors* [3–5]. The high density of social interactions enhances the division of labor, which is traditionally considered as responsible for the economic productivity of cities [6]. Nowadays, the relationship between the social and economic aspects of cities is intensively studied within the growing field of the science of cities [7], drawing on a broad palette of quantitative and computational methods and insights from complexity science [5].

It is indisputable that the ancient cities share many key futures with their modern counterparts. For instance, recent research on urban scaling has identified the same scaling properties in the relationship between a city’s area and its population size [4]. These similarities suggest that lessons from the past can help us navigate contemporary urban systems and guide their development. Data collected by historians and archeologists are particularly valuable because they allow us to study the processes of rise and decline of entire civilizations, including the persistence and sustainability of their urban systems in the long-term [1].

One of the intriguing questions that concerns the dynamics of cities across different historical contexts touches on the relationship between the specialization and diversity of a city on one hand and its resilience and productivity on the other [8]. Following [9], we define a city’s specialization as the extent to which occupations within a single urban area are accumulated in one particular industry sector—hence sectoral specialization. Diversity, on the other hand, is determined by the extent and equality with which employment is distributed across multiple industry sectors. While high sectoral specialization results in a higher efficiency and greater economic productivity of a city as a whole, it also reduces urban resilience in light of shock or change [8, 10–12]. Detroit is a modern example whose focus on the car industry contributed to its economic eclipse in the end of the twentieth century [13]. The “Dead cities” of Syria offer an ancient example of peasant towns in the Roman East whose prosperity depended solely on olive oil production for export and collapsed once the Arab incursions disrupted international trade [14]. High sectoral diversity in cities, where multiple domains of production co-occur, fosters innovation, limits exposure to risk, and increases resilience.

It is now generally accepted that the socio-economic performance of the Roman Empire during the first two centuries CE was higher than during the periods which preceded and which followed [15–17]. Some scholars even suggest for a few generations, the Empire was able to escape the “Malthusian Trap” and to witness a short period of “Efflorescence” [18]. The economic performance of the Empire is documented by reference to various archeological proxy data, such as ancient Mediterranean shipwrecks [19, 20], deposits of animal bones [16, 21], traces of pollution Greenland’s ice cores [22–25], or the changing aggregated capacity of fish-salting factories across the Empire [26]. Of course, the details of the picture differ from dataset to dataset. It is not only because of the varying quality and temporal resolution of the data, but also because they capture different aspects of the socio-economic development of the Empire: while the temporal distribution of shipwrecks perhaps informs us primarily about the integration of trade, the animal bones and fish-salting factories tell us mainly about consumption [27]. This article expands such research by focusing on another aspect of socioeconomic development: division of labor, a phenomenon closely associated with urbanization. Other quantitative studies of division of labor in the cities of the Roman Empire already exist [28]. However, they offer a synchronic view. The novelty of our study is that it offers a diachronic overview of the division of labor from 50 BCE to 350 CE.

To assess the dynamics of labor division in the Roman Empire, we first employ computational text analysis methods to identify and extract all instances of occupations in a dataset of ancient Latin inscriptions, using an newly aggregated extensive list of Latin occupational titles [29–31]. Second, we explore how the occupational data are distributed across various industry sectors (e.g. Metal-Working, Food-Production etc., cf. [32]) and how these sectors are represented in urban contexts of different population sizes. Subsequently, we analyze the temporal distribution of the occupational data. Finally, we focus on individual cities as units of analysis and use the inscription data to gauge to what extent each city is relatively specialized or diversified in different industry sectors. Assessment of diversity and specialization in cities at the scale of the Western Roman Empire has to our knowledge not been attempted, and our study thus represents a first undertaking in this direction.

Inscriptions survived in large quantities, containing valuable information about cultural norms, social structure and demographic distribution of people involved in commissioning inscriptions. The rate of survival of inscriptions certainly poses a challenge for any quantitative analysis as inscriptions on durable materials such as stone or metal were often reused or repurposed. Yet the surviving 500,000 Latin texts contain a substantial amount of information, ranging from textual references to Roman society and economy to temporal and geospatial indicators [33–35]. Inscriptions are traditionally considered to be an urban phenomenon, as most are found in the vicinity of settlements or military installations and thus represent the most accessible proxy for the study of division of labor in the ancient world [36–38]. It is widely accepted that the cultural habit of producing inscriptions in the Roman world first became widespread in large cities and only later and on a minor scale has been adopted elsewhere, namely in small towns and in the countryside [28, 34]. It is only now that we can study the epigraphic collection as a whole and test qualitative propositions—such as the delay in the adoption of epigraphic production in cities of smaller sizes—quantitatively.

A shared trait among the Latin inscriptions is the tendency to advertise someone’s social status. Public declaration of one’s achievements permeates this medium, focusing especially on political, administrative, military function, or the status of citizenship. Status display flourished particularly in the urban environments which offered higher social mobility [34, 38, 39]. Most inscriptions also carry some statement of identity, such as personal name, geographic origin, social group or occupation. The conception of what comprises a profession has changed since Antiquity as has the value assigned to the profession. Studies of epigraphic sources, however, assert that professional occupations carried prestige and social status in Antiquity [40]. One study of inscriptions from the city of Rome shows that over 60% of all texts mentioning occupations were commissioned by freed-men and slaves, who proudly signal their accomplishments and elevated social standing despite originally low social status [41]. According to this study, inscriptions preferentially mention socially prestigious occupations such as administrative and managerial jobs that require skills, education, or training. These contrast with menial activities, such as food provisioning, deliveries or building supplies. We therefore expect to see an inflation in the skilled positions in the epigraphic data, namely in the administrative and managerial sector, services, and in general in the tertiary sector, when compared to unskilled positions in agriculture, building or transport.

## Materials and methods

Data preparation consisted of extracting and categorizing occupation data from the text of inscriptions and linking inscriptions with the buffers of ancient urban zones. This way we generated a dataset of inscriptions with attributes characterizing their urban context and a dataset of cities with attributes capturing occupational information as well as urban characteristics.

### Epigraphic dataset

The study is based on a recently published dataset of Latin inscriptions of the Roman Empire (LIRE, *N* = 136,190) [42]. LIRE is an aggregate of inscriptions from two public epigraphic databases: Epigrafik Datenbank Clauss-Slaby (EDCS, *N* = 500,618) and Epigraphic Database Heidelberg (EDH, *N* = 81,476). However, LIRE contains only those inscriptions from EDCS and EDH that satisfy the following criteria: (1) records contain valid geospatial coordinates, (2) coordinates fall within the boundaries of the Roman Empire at its largest extent in 117 CE, (3) metadata contain the most plausible date of creation (typically having form of a temporal interval), and (4) date of an inscription intersects with the timespan of the Roman Empire (arbitrarily set to 50 BCE through 350 CE).

The content of EDCS and EDH partially overlaps. The LIRE dataset contains 83,482 inscriptions originating exclusively from EDCS, 3,907 inscriptions originating exclusively from EDH and 49,916 inscriptions shared by the two resources. The records from EDCS are accompanied by 29 attributes containing metadata while the records from EDH have 74 attributes. Some of the attributes overlap and can be easily mapped between the two resources, like numerically expressed geographic coordinates. In the case of other attributes, like the type of inscription, the mapping is much less straightforward as the two databases use different classification schemes (see [43]). For inscriptions covered by both datasets, LIRE inherits attributes from both collections, while giving preference to attribute values from EDH, which is generally considered to be a more carefully curated resource [44, 45].

To some extent, EDCS and EDH differ in their overall spatio-temporal coverage. EDCS is more comprehensive, covers a broader spectrum of epigraphic production and also systematically reflects the magnitude of inscriptions generated by the city of Rome. EDH, on the other hand, contains only a sample of inscriptions from the city of Rome, but offers a uniformly distributed, high-quality and metadata-rich dataset covering the western Roman provinces (for a detailed comparison, see [33]). By combining these two resources and by filtering the records according to the above specified criteria, the LIRE dataset provides a more representative overview of surviving Latin epigraphic production from the times of the Roman Empire, rather than the two databases treated independently.

The fact that we focus on Latin inscriptions implies that our analysis is mainly applicable to the western part of the Empire, where Latin was the dominant language [34, 46, 47]. To conduct an analysis of Empire-wide trends, we would need to also include inscriptions written in the Greek language. Although most Greek inscriptions have been digitized and are searchable online, they are not yet available in a form that would make quantified computational analysis possible [45, 48].

### Roman cities, urban contexts and western provinces

To enrich the inscription data with urban attributes and nearest city population size, this study employs the database of cities collected by Hanson as the most comprehensive analysis-ready dataset [49]. The database lists 1,388 cities with metadata, including geographic coordinates, extent of the inhabited city area, etc. To estimate the population size of these cities, we employ the function from [50], which calculates population size of each city on the basis of its estimated inhabited city area, relying on the so-called densification effect. The inhabited city area is typically approximated or inferred from the area enclosed by the detected city walls. For cities where this area is unknown, we make a conservative estimate of 1,000 inhabitants. In total, this procedure results in the urban population of the Roman Empire equal to 10,214,337 inhabitants.

Drawing on the population estimates, we divide the cities into three groups: *large cities*, i.e. cities with population estimate equal to- or higher than 30,000 inhabitants (*N =* 68, total population = 4,641,352, *medium cities*, i.e. cities with population estimate equal or higher than 5,000 inhabitants but smaller than 30,000 (*N =* 337, total population = 4,005,250), and *small cities*, i.e. cities with population lower than 1,000 inhabitants (*N = 983*, total population = 1,513,068). By default, in most of our analyses, we include Rome (population = 923,313) among large cities, together with significantly smaller cities, such as Lepcis Magna (population = 153,722), Carthago (population = 102,170), or Londinium (population = 36,851). However, in some cases we also offer supplementary analyses either treating it separately or excluding it completely (especially in the case of correlational analyses).

In the next step, we focus on the neighborhood of these cities which we define by circular euclidian buffers with a 5 km radius. The 5 km distance roughly corresponds to one hour of walking, what has been observed as a mean traveling time per day cross-culturally [51]. It has been also claimed that most agricultural activities of a settlement usually terminate at the distance of 5 km [52]. Using these buffers and intersecting inscription locations, we enrich the dataset of cities with information from all inscriptions in their neighborhood. Simultaneously, we extend the dataset of inscriptions by attributes containing information about its nearest city and the urban context it represents (*large*, *medium*, or *small*). In cases where an inscription is in the buffer of more than one city, the larger one is preferred. Inscriptions not covered by these buffers were classified as coming from rural areas.

Drawing on the list of western provinces from [53], we further differentiate between cities and inscriptions from western and eastern provinces. Given that Latin was predominantly spoken within the western provinces of the Empire [34] and that our epigraphic dataset contains only Latin inscriptions, some of our analyses are narrowed to inscriptions and cities from the western part of the Empire, for which our data is more representative. From the 1,388 cities in the dataset, 889 have been classified as western, which correspond to 64%. From the 136,190 inscriptions in the LIRE dataset, 125,640 have been located within the western regions, which is more than 92%.

### Occupations and occupational categories

To gauge ancient Roman labor division on the basis of inscriptions, we manually created a list of Latin terms for occupations and their known spelling variants (N = 882) (see S2 Data). We then programmatically developed a full declension paradigm for these terms. We have also created full declension paradigms for occupation names consisting of more than one word. The declined versions of all terms and their combinations were used to search for individual occupations within the clean text of inscriptions. This way we were able to proceed without the necessity to lemmatize the corpus of inscriptions as a whole, a procedure naturally prone to mistakes, especially when applied to textual data with unstable syntax and missing sentence division and with missing models trained specifically on epigraphic corpora.

As a result, we obtained a list of all mentioned occupations for each inscription that allowed us to calculate the total number of occupations of any type within any group of inscriptions and weight it by the total number of inscriptions or words within the group (i.e. their relative frequencies).

The calculation of frequencies in our case is based on data with highly positively skewed distributions, with the vast majority of inscriptions containing no mention of occupations. Being aware of this, we employ the bootstrap test proposed in [54] to decide whether there is a significant difference in the relative frequency of occupations between any two groups of data. This bootstrap test is designed to compare word frequencies between two text corpora, including those with extremely skewed distributions [35]. For each run of the test, we have two groups of inscriptions: group 1 and group 2. Within each bootstrap cycle, 1,000 inscriptions are randomly sampled from group 1 and 1,000 inscriptions are randomly sampled from group 2 (the sampling is with replacement, i.e. one inscription might be chosen repeatedly during the sampling). Subsequently, for both samples, occupations are counted. Once this process is finished, we calculate the proportion of bootstrap cycles for which the value for group 1 is higher than the value for group 2. This proportion is subsequently employed to calculate the *p-value* using the following equation:

$$p=\frac{1+2N\times p1}{1+N},$$ (1)

where *N* is the number of bootstrapping cycles and *p1* is the proportion of bootstrap cycles for which the value for group 1 is higher than the value for group 2 or vice versa (see [55]).

Each occupation on the list of Latin terms for occupations has been also classified according to two classification systems: The Historical International Standard Classification of Occupations (HISCO) categories (*N* = 10) [56] and a classification scheme developed by Harris for occupations from classical Athens (*N* = 14) [32]. HISCO represents a standard classification system for historical occupations divided into 10 major group categories, such as Professional, technical and related workers, Administrative and managerial works or Clerical and related workers. The HISCO groups are well suited to capture the vertical stratification of a society, with individual categories referring to specific types of work activities, group 0 representing the jobs requiring specialized training and high skill set level, while group 9 represents mostly manual low- and unskilled labor [57]. Although the HISCO categories were originally designed to capture occupations from the Early Modern period, they were applied in a synchronic study of occupations in the Roman world, despite some category incongruencies [58–60]. On the other hand, Harris’ categories [32] focus on individual industry types, such as Metal-Working, Food-Production or Clothing, which makes them more suitable to capture relative specialization and diversity of cities (*sensu* [9]). They reflect the reality of ancient occupations, especially the ambiguous producer/trader distinction, but do not provide skill-level classification [58, 61]. In order to adapt Harris’ division to the Roman world, we have added five categories to describe sectors not detected in Classical Athens, such as Managerial, Water-management, Glass-working, Death-care (funerary services), and Unclassified (N = 19).

### Modeling temporal uncertainty

Diachronic analysis of inscriptions is complicated by the uncertainty accompanying their date, ie. the assumed year of their creation. There are inscriptions which are dated quite precisely into a singular year or two, usually because they refer to specific historical events (e.g. someone’s consulship). But there is also a substantial amount of inscriptions which lack such references and might be dated only very roughly, with reference to half-century, century, or even longer historical periods. This varying precision might be numerically expressed by an interval delimiting the earliest and the latest year during which an inscription is assumed to be produced. In the case of LIRE, almost 25% (*N* = 33,920) of all inscriptions are dated by interval with duration equal to 100 years and almost 13% (*N* = 17,658) of all inscriptions by interval with duration equal to 200 years (see S1 Fig, S1 Data).

To deal with temporal uncertainty in our data, we adopt a Monte Carlo simulation (MCS) approach introduced by [62] and implemented in [63]. This approach allows us to analyze temporal trends without excluding the broadly dated data points from the dataset; we can also combine the temporal analysis with more in-depth measurements. To each inscription, we first assign 1,000 random dates within its dating interval. The random assignment follows uniform distribution, which implies that an inscription dated to the second century CE (ie. with the dating interval from 101 CE to 200 CE) is assumed to have equal probability to be produced at any year during the century (e.g. 104 CE, 149 CE, or 188 CE); an inscription dated to a single year is 1,000 times assigned to the same year. The random dates are subsequently recombined into 1,000 time-series simulations, with each time-series simulation containing one previously generated date for each inscription. The time-series simulations are constructed either for the dataset as a whole or for some preselected subsets of the data (e.g. by urban context, geographic provenance or type of the inscription).

The temporal distributions of the time-series simulations are then analyzed in several different ways. First, we explore the data visually by means of cumulative gaussian kernel density estimate (KDE) plots, with each time-series simulation represented by one KDE line. The bandwidth of the KDE is calculated automatically on the basis of Scott’s rule [64]. This allows us to visually explore temporal trends in the data in respect to specific historical events or periods. Greater spread in the KDE lines implies a greater amount of temporal uncertainty in the underlying data, which constrains the possibility to make strong inferences about temporal trends in the data.

As an alternative method, we analyze the time-series simulations using prespecified timeblocks. We work either with 50-years-long timeblocks or with time blocks based on reigning dynasties. This allows us to combine the analysis of temporal distribution of inscriptions with an analysis of their language content and to statistically compare the data from one period to another. The method works as follows: For each time-series simulation, the whole dataset of inscriptions is divided into subsets based on prespecified timeblocks. The subset of inscriptions within each timeblock is slightly different from one time-series simulation to another, because the random dates assigned to individual inscriptions often come from a dating interval longer than one timeblock. Thus, in one time-series simulation, an inscription dated to the second century CE (101 CE– 200 CE) is included among inscriptions from the first half of the second century, in another simulation it is included among inscriptions from the second half of the second century. Next, within each timeblock, the subset of inscriptions is used for further measurement, e.g. the total number of words or a frequency of certain words. The robustness of the results might then be evaluated by comparing them across the simulations. Larger variance of the measurement within one timeblock across the simulations implies higher extent of temporal uncertainty in the underlying data and therefore mitigates the possibility to make strong inferences concerning differences between timeblocks.

The timeblock analysis is further combined with the bootstrapping method described above. In this case, each time-series simulation is used to retrieve one bootstrap sample per timeblock.

Finally, in some cases we also employ a two-sample Kolmogorov-Smirnov test to compare distributions of any two time series. This way we compare the temporal distribution of all inscriptions containing mentions of occupations with a random sample of inscriptions of the same size. We repeat this procedure for multiple time-series simulation combinations and then report the mean values.

### Sectoral specialization and diversification of cities

After the exploration of labor division in the Roman Empire through time, we quantify the composition of occupational activities linked with individual cities. To measure specialization and diversity of ancient Roman cities, we constrain our attention to cities and inscriptions from the western part of the Empire, for which the epigraphic dataset is considered to be more representative (889 cities and 125,640 inscriptions). Since the dataset of cities and the dataset of inscriptions have been mapped on each other, for each city we can easily obtain a list of all occupations appearing on inscriptions from its neighborhood. These lists of occupations for each city are finally used to measure their specialization and diversity.

Drawing on standard approaches [9], we have explored several different ways to measure specialization of cities. The starting point is to measure the employment share of each city’s largest sector. In our case, the sectors are substituted by occupational categories from Harris (see above). Thus, for each city (*i*), we first count the number of occupations in each occupational category (*j*) and its share (*S*) in the total number of occupations from a given city. Subsequently, we extract the share of the largest sector, defining absolute specialization index of each city (*ZI*_*i*_) such as:

$${ZI}_{i}={max}_{j}(S_{ij}),$$ (2)

Unfortunately, our dataset includes a number of cities for which we have only one occupation documented, and consequently also only one occupation category. In such cases, *ZI*_*i*_ returns unrealistically high specialization values. For instance, a city with one occupation in total has a higher *ZI*_*i*_ value than a city with ten occupations in total with nine of them from one sector. But we would hesitate to say that the first one is more specialized than the second one. Instead, we recognize that the occupational data extracted from the inscriptions capture only a very small sample of occupations from a given city. To deal with this limitation of our occupational data, we modify the *ZI* score by multiplying it by the natural logarithm of the total number of occupations in a given city (*N*_*i*_). Thus, we define weighted specialization index (*ZIw*_*i*_) such as:

$${ZIw}_{i}={max}_{j}(S_{ij})\times log(N_{i})$$ (3)

For our dataset, this measurement appears to be a useful indicator of a city’s specialization. However, it is not sensitive to the fact that the sectors are distributed unequally over the occupational data and that some sectors have locally a bigger share because they are globally more widespread. To control for this factor, we have further implemented a weighted relative specialization index (*RZIw*_*i*_), which divides the share of each sector in the city by its dataset-wide share:

$${RZIw}_{i}={max}_{j}(S_{ij}/S_{j})\times log(N_{i})$$ (4)

To measure diversity, we use the inverse of Hirschman-Herfindahl index, summing for each city over all sectors the square of each sector’s share in local employment. Thus, the diversity index is calculated as follows:

$${DI}_{i}=1/\sum_{j}S_{ij}^{2}$$ (5)

Again, as in the case of specialization, we have to consider that the score returned by this measurement is to some extent dependent on the size of the sample data, this time in an opposite direction: A city with more occupational data is more easily identified as having higher diversity than a city with a small amount of occupational data. To control for this, we additionally introduce a weighted variant of the same measure, dividing the *DI* score by the logarithm of the number of occupations:

$${DIw}_{i}={DI}_{i}/log(N_{i})$$ (6)

## Results

### Inscriptions and occupations across urban contexts in the Roman Empire

As described in the Methods section, we mapped each inscription in the LIRE dataset on a city from the dataset of cities. These cities have been already divided into three categories: large, medium, and small. From the 136,190 inscriptions in the LIRE dataset, 43,062 have been mapped on a large city, 34,062 inscriptions on a medium city, and 25,847 inscriptions on a small city. The remaining 33,229 inscriptions have been classified as rural, since they are located out of the buffers of all cities.

In total, we extracted 5,222 instances of 387 unique occupations dispersed over 4,161 inscriptions across the Empire. The most common occupation is *curator* (*N* = 1,062), followed by *faber* (*N* = 566) and *medicus* (*N* = 252). At the opposite end, there are 137 occupations occurring only once (for an overview of all identified Latin occupations, including their counts, translation and occupational category, see S3 Data).

Occupational terms are most often mentioned on funerary inscriptions, followed by votive and owner/artist inscription types, which also represent the largest group of inscriptions. When we measure the frequency of occupational terms per 1,000 words, honorific inscriptions mention occupations the most often (for details see S2 Fig). Honorific inscriptions tend to list all personal achievements and official positions. With the professional pride increasing, the high frequency of occupational terms in honorifics is not surprising.

**Fig 1:**
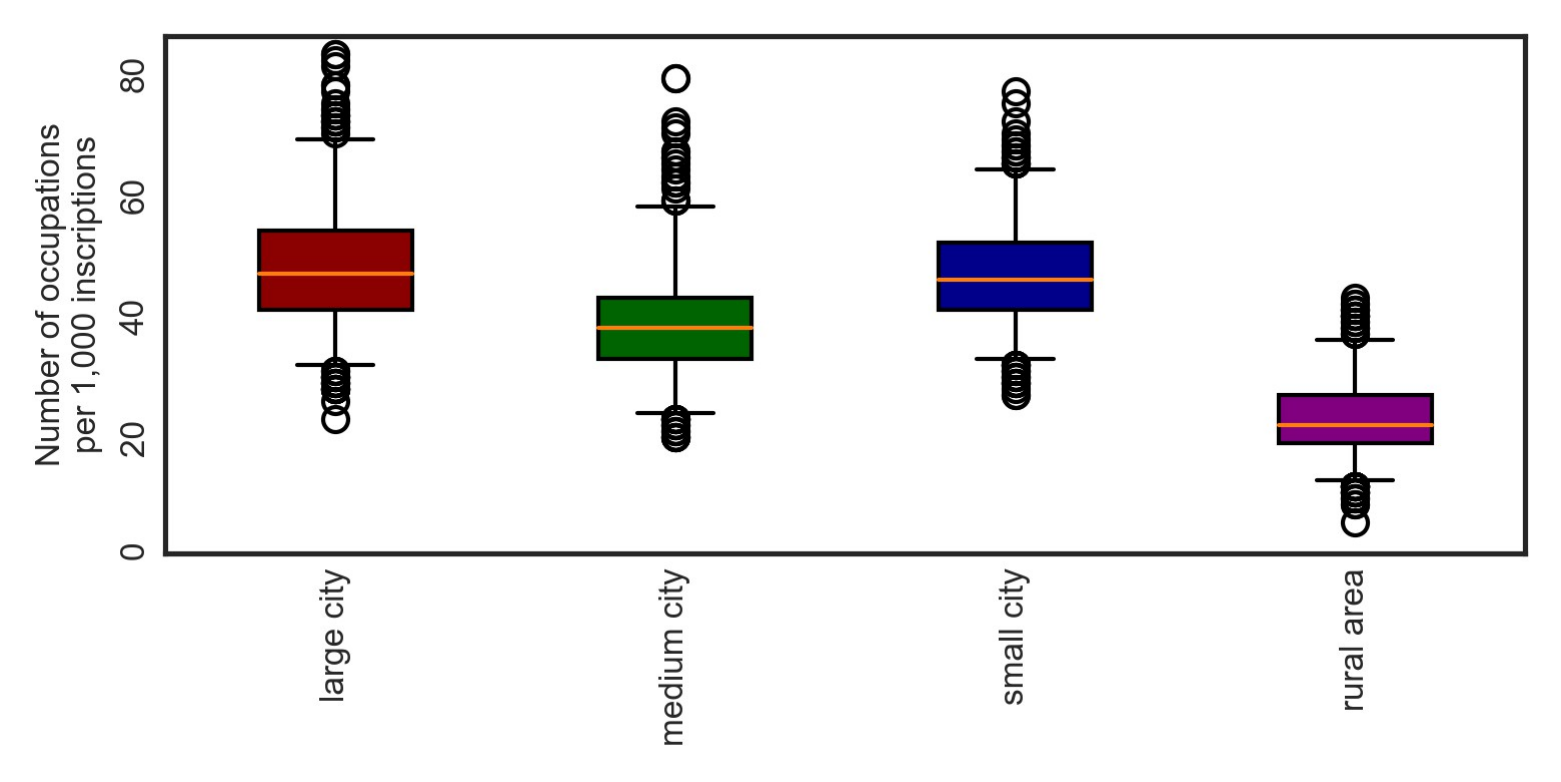
Occupations across urban contexts. The plots depict results of 1,000 bootstrapping cycles. Boxes correspond to inner quartiles and whiskers to 95% confidence intervals.

To some extent, the frequency of occupation terms depends on the urban context (see Fig 1). A random sample of inscriptions from large cities contains on average more occupation terms than a random sample of inscriptions from medium cities. A random sample of inscriptions from small cities contains on average more occupation terms than a random sample of inscriptions from rural areas. However, a random sample of inscriptions from medium cities on average does not contain more occupation terms than a random sample of inscriptions from small cities (see S4 Data). Employing the bootstrap test, we see that despite an obvious trend, most of the differences are not statistically significant, with a few notable exceptions: First, there is a statistically significant difference between the mean count of occupation terms in a random sample of inscriptions derived from all city contexts combined and the count of occupation terms in sample from rural areas, and second, there is a statistically significant difference between the count of occupation terms in a sample from small cities and the count of occupation terms in a sample from rural areas. In other words, the frequency of occupation terms is significantly higher on inscriptions from cities than on inscriptions from rural areas. It appears that the frequency tends to be even higher in larger cities, but using our data, we are not able to capture this difference statistically. Being aware of the peculiarity of the city of Rome, we also rerun the analysis treating it separately from other large cities (S3 Fig). Surprisingly, this supplementary analysis suggests that the high frequency of occupational terms on inscriptions from large cities is not driven by Rome in particular, but rather by other large cities. This can be explained by a large proportion of inscriptions from the period ca. 300 CE exclusively from the city of Rome, which reflect the advent of Christianity and which are devoid of occupational terms.

**Fig 2:**
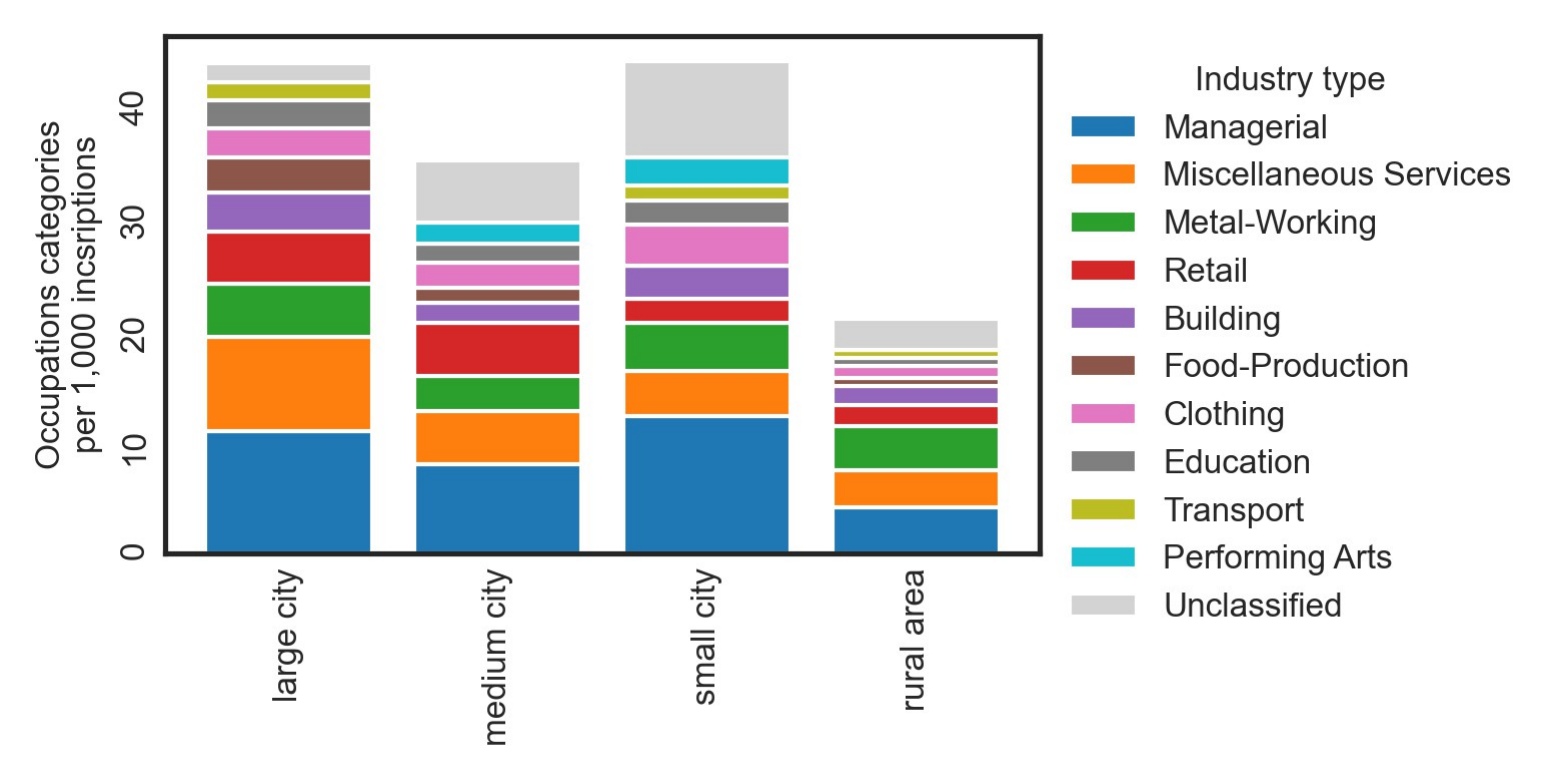
Occupations by industry type across urban contexts. The bars express the cumulative frequency of individual occupation types per 1,000 inscriptions.

Next, we made an exploratory analysis comparing the distribution of occupations grouped by industry types. For this analysis, we calculated the number of individual occupation types per 1,000 inscriptions and plotted the results by means of a stacked bar plot (Fig 2). The analysis reveals that in large cities the biggest proportion of occupations fall in the Managerial (such as *curator* (an overseer or manager), *horrearius* (a superintendent of a storehouse)) and Miscellaneous Services sectors (such as *medicus* (a surgeon), *mensor* (a land surveyor), or *unctor* (an anointer)). In comparison to other urban contexts, Miscellaneous Services appear to be most common in large cities where the demand for specialized skills and services is higher [39]. Education and Transport, which are overall much less widespread categories, are also most common in large cities. Looking at medium and small cities, we again see the Managerial sector widely represented, especially in small cities. Medium cities further reveal a noticeable number of occupations in the Retail sector. The rural areas do not reveal to be characterized by any individual sector in particular. We might be surprised by the low number of occupations in the Food-Production sector, which also covers occupations associated with agriculture. According to some estimates, 80–90% of the population worked in the agriculture and Food-Production sector [39]. But we should realize that farming was not considered a profession *per se* and the occupational pride connected with jobs in agriculture and other food production might have been relatively low, leading to its limited epigraphic footprint [40]. Again, we repeated the analysis treating Rome separately from other large cities (S4 Fig). We see that the high prominence of Miscellaneous Services and other categories is also visible in large cities excluding Rome.

We have also rerun the same analysis while employing the occupation classification system of the HISCO major groups (see S5 Fig). We see that overall the most widespread category is Production and related workers, transport equipment operators and laborers. Behind that, we observe that large cities dominate in categories like Service workers, Sales workers, or Clerical and related workers. Thus, again, large cities are characterized by occupations from what we could call a tertiary sector. In that respect, it appears that our findings are not very much dependent on a particular classification scheme.

All HISCO major groups have a numerical code ranging from 0 to 9 expressing the level of required skills to perform the job (group 0 represents jobs requiring the most specialized training and high skill set level, while group 9 represents mostly manual low- and unskilled labor). Thus, the mean value of HISCO groups of all occupations within an urban context informs us about the extent of skilled labor within the urban context in the way that lower average value indicates a higher extent of skilled work. We find the lowest value in large cities (*M* = 4.28), followed by medium cities (*M* = 4.74), small cities (*M* = 4.93) and rural areas (*M* = 5.36).

Taken together, these findings are in agreement with our general expectation that as we move from rural areas toward large cities, there is an increasing number and variation in occupations. Even more importantly, there is also an obvious shift toward the tertiary sector, being more broadly represented in large cities, namely by Miscellaneous Services, Education, and the Managerial sector. Even though the sizes and the level of available information is incomparable, sector ratios bear resemblance to modern cities. The service sector is most pronounced in the large cities, while medium-sized and small cities focus on manufacture (such as textiles, metal or food) [65]. Interestingly, the proportion of agricultural workers (Food-Production sector) in large cities is higher than in rural areas or small cities. Food-producers enjoyed higher status in cities whose provisioning was a challenge requiring mass production [61].

### Temporal distribution of inscriptions and occupations

**Fig 3:**
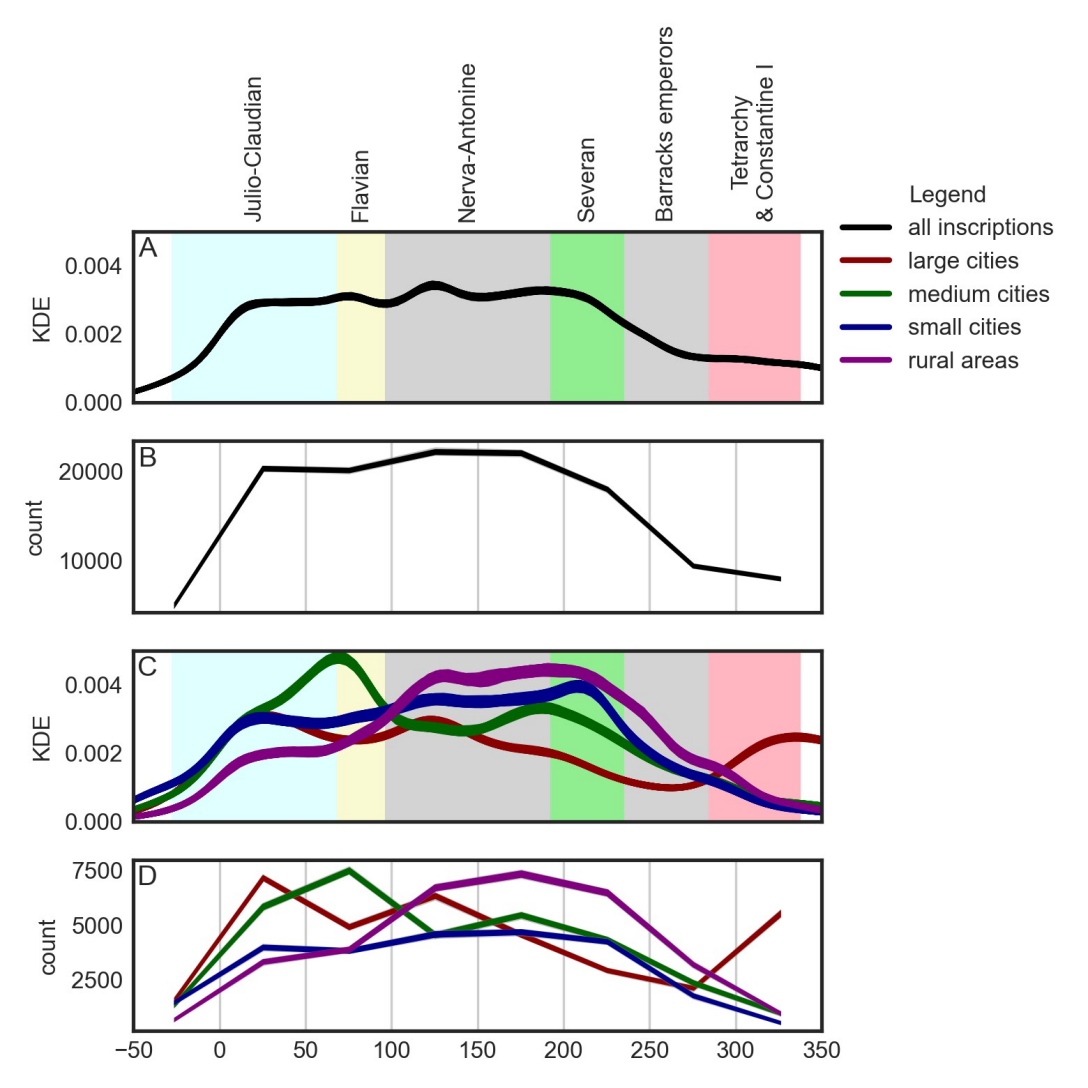
Temporal distribution of inscriptions by urban context.

Before we delve deeper into an analysis of temporal distribution of occupational data, we first look at temporal distribution of the LIRE epigraphic dataset as a whole. Fig 3 depicts the temporal distribution of inscriptions employing two different visualization methods (see Methods, Modeling temporal uncertainty): a method based on kernel density estimation (subplots A and C) and a method based on uniform timeblocks. On all four subplots, the curves are based on 1,000 Monte Carlo time-series simulations, assigning to each inscription a random date within its dating interval. While the KDE method is more sensitive, we complement it here by the method based on uniform timeblocks, since it allows us to compare absolute numbers of inscriptions per timeblocks. Further, for historical contextualization, on the background of subplots A and C, we see individual reign dynasties or specific periods in the history of the Roman Empire.

When we look at the subplots A and B, we see that—according to this dataset—the production of inscriptions takes off in the middle of the Julio-Claudian era and the volume remains high until the Severan dynasty. The absolute peak in the production of inscriptions falls into the reigns of emperors Trajan (98–117 CE) and Hadrian (117–138 CE) during the Nerva-Antonine dynasty (96–192 CE). We further observe that there is a long-term decrease in the epigraphic production starting with the Severan dynasty and continuing throughout the crisis of the third century. Subsequently, the decrease continued during the Tetrarchy and the reign of Constantine (285–337 CE), but at a slower pace.

However, when we look at the lower two subplots (C and D), the overall picture becomes more complex, as we see that the dynamic differs substantially depending on urban context. Inscriptions from the urban context of large cities peak in number in the first half of the first century, while inscriptions from medium cities reach maximum values approximately half a century later, in the second half of the first century CE. In the case of small cities, we observe a linear increase through a two-century-long period starting in the first half of the first century CE and culminating during the first half of the third century CE, i.e. two centuries after large cities. The temporal distribution of inscriptions from rural areas appears to be most similar to the temporal distribution of inscriptions from small cities, with the highest values spanning the first half of the second and the first half of the third century CE.

The previously described decline in epigraphic production in the early third century deserves a more nuanced interpretation, too. The number of inscriptions in large and medium cities decreases well before the advent of the third century; in the case of large cities, before the middle of the second century. The epigraphic production in small cities and rural areas, however, flourishes well into the third century. The production in small cities, in fact, peaks during the early decades of the third century.

Taking these observations together, our findings support a common claim that the “epigraphic habit” first became widespread in large and medium cities and only subsequently has been adopted by small cities and in rural areas, approximately one century later [28, 34]. Accordingly, the decline started first in large and mid-sized cities, to be later followed by small cities and rural areas.

### Temporal distribution of occupations

As we have already seen, the occupational terms are distributed unequally through the epigraphic data, being more frequent on inscriptions from cities than on inscriptions from rural areas. In what follows, we offer a similar analysis with respect to temporal distribution of occupations.

**Fig 4:**
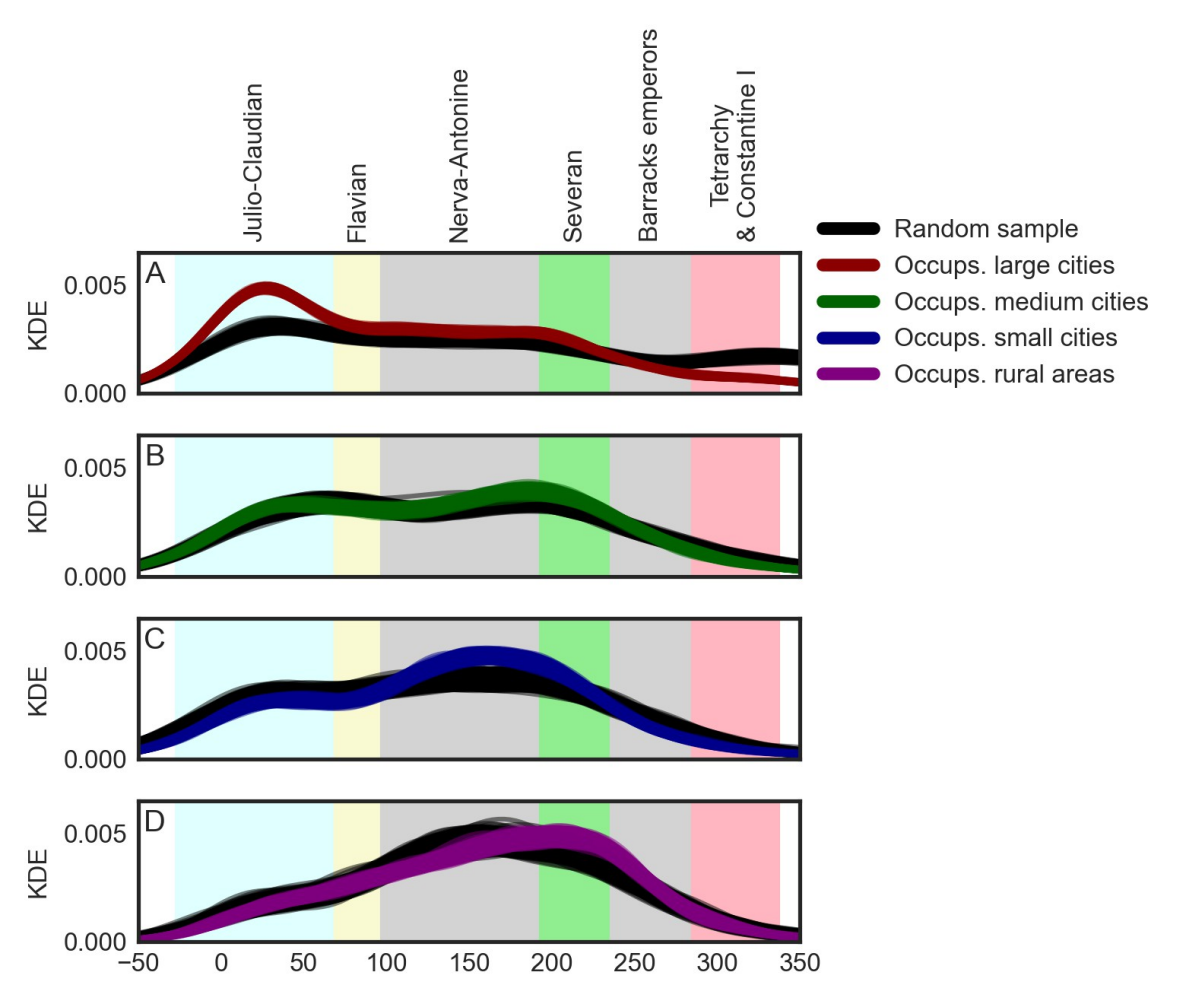
Temporal distributions of occupations using the KDE method and control samples.

On Fig 4 we see how the temporal distributions of inscriptions naming occupations deviate from temporal distributions of random samples of inscriptions. In each case, the random sample is formed by the same number of inscriptions and of the same urban context as the corresponding subset of inscriptions naming occupations. The types of inscriptions are proportionally represented, too. For instance, in large cities, there are 1,598 inscriptions naming at least one occupation. 813 of them are classified as epitaphs. The corresponding random sample has the same size and composition of types of inscriptions and is used to control the results. The target subset of inscriptions naming occupations is recombined to produce 1,000 time series. Accordingly, the random sample is generated 1,000 times, each time producing one time series. Thus, for each urban context, there are 1,000 time series representing the target subset of the data and 1,000 time series representing the control samples.

Upon a visual inspection of Fig 4, we see that across all four urban contexts the temporal distributions of inscriptions naming occupations slightly deviates from the temporal distributions of the control samples. However, employing the Kolmogorov-Smirnov test, we see that the deviation of the target subset from the control sample is statistically significant only in the case of large cities (avg. *KS statistic* = 0.1891, avg. *p*<0.001) and small cities (avg. *KS statistic* = 0.0713, avg. *p* = 0.039). In the case of large cities, as seen on subplot A, the difference between the target subset and the control sample is most remarkable during the first half of the first century, when the kernel densities of the time series based on the target subset of the data are well above the curves produced by time series of the control samples. This rise in the frequency of occupations is contemporary with the labor-intensive Julio-Claudian building program, which gave rise to monumental Rome and Italian cities, employed a large number of people, and likely prompted various new professions [66].

**Fig 5:**
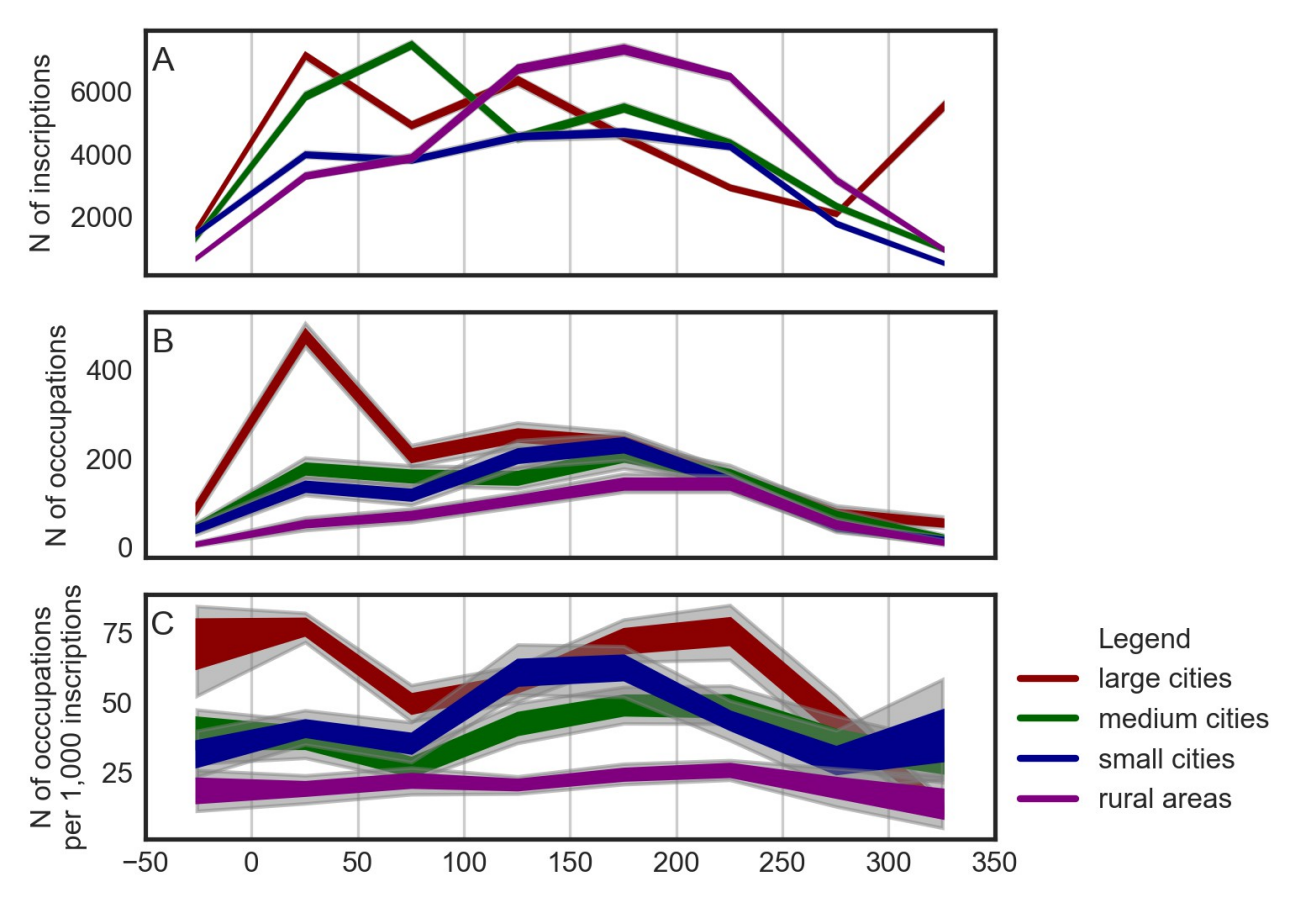
Temporal distributions of inscriptions and occupations using uniform 50-years-long timeblocks. A—temporal distributions of inscriptions; B—temporal distributions of occupations; C—temporal distribution of occupations per 1,000 inscriptions. Gray color represents data from the underlying time series out of the 95% confidence interval.

Fig 5 allows us to inspect this last observation a bit further. The subplot A is the same as subplot D on Fig 3 and we include it here for reference only. The subplot B depicts counts of occupational terms. Again, here we can easily spot the remarkably high number of occupation terms from inscriptions from large cities in the first half of the first century CE. To put this observation into perspective, in the LIRE dataset as a whole, there are (depending on the time-series simulation) between 20,064 and 20,529 (*M* = 20,922.57) inscriptions dated to the first half of the first century CE. From these inscriptions, approximately 35% have been located in large cities and 33% have been located in the city of Rome alone. In sum, the inscriptions from the city of Rome from the first half of the first century CE represent approximately 5% of the whole LIRE dataset. In these 5%, we have identified between 483 and 588 occupation terms (*M* = 546.26), depending on the sample and its source time series. It is more than 10% of all occupation terms (*N* = 5,222) identified across all periods and urban contexts.

The subplot C is based on a combination of data from subplots A and B. It depicts the frequency of occupational terms in random samples of 1,000 inscriptions from a given urban context. First of all, while in the case of large and small cities the frequencies of occupational terms visibly differ from timeblock to timeblock, in the case of medium cities and rural areas, the frequencies tend to be almost constant. For instance, there are almost twice as many occupational terms per 1,000 inscriptions from small cities in the first half of the second century CE (*M* = 60.93), than during the preceding timeblock (*M* = 35.49). The bootstrap test indicates that this difference is statistically significant (*p* = 0.03), but the effect size is relatively small (Cohen’s *d* = 0.07).

The subplot C also sheds new light on the above discussed temporal distribution of occupations on inscriptions from large cities, peaking during the first half of the first century (see subplot B). We see that the frequency of occupation terms on samples of inscriptions from the second half of the second century CE is approximately the same as the frequency on samples from the first half of the first century CE. This serves us as a warning against putting too much emphasis on observations based on raw counts as seen in subplots A and B: raw counts have to be always compared with frequencies.

The last observation based on the temporal distribution of occupations we want to elaborate is concerned with the overall decrease of occupation-term frequencies between the first and second half of the third century CE. We see a decrease of the raw counts of the occupation terms (subplot B), but also their relative frequencies (subplot C). This decline is visually detectable across all four urban contexts. However, according to the bootstrap test, only the large cities provide statistically significant results (*p* = 0.03, Cohen’s *d* = 0.09), probably due to the temporal uncertainty of the underlying data. Upon a visual comparison of subplots A and C, the decline in occupation-term frequencies may to some extent correlate with the overall decline in the number of inscriptions in the dataset. However, the decline in the number of inscriptions starts first and the decrease in the frequency of occupational terms follows with certain lag. This might be associated with the complex relationship between economic productivity and resilience of cities on the one hand and their sectoral diversification and specialization on the other.

### Sectoral specialization and diversification of cities

In the previous sections, we have analyzed occupational data from the entire Roman Empire across four types of urban contexts. We now zoom to the level of individual cities and analyze epigraphic data associated with each one to assess the extent to which individual cities were specialized or diversified and whether the different structures bore them any benefits. To be comparable with other attributes of the cities (esp. their population size estimates), we narrow our analysis to cities from the western part of the Empire, where the Latin inscriptions serve as a more representative proxy.

From the 1,388 cities in the dataset, 889 are located within the western part of the Empire. 723 of the western cities are associated with at least one inscription, and 360 cities are associated with inscriptions containing at least one occupational term. In total, these 360 cities accommodate 3,250,628 inhabitants, which represent approximately 60% of the estimated urban population of the western part of the Empire and 32% of the estimated urban population of the Empire as a whole. The subsequent analysis focuses on this subset of 360 cities.

Concerning the epigraphic data, 125,640 (92%) of the 136,190 inscriptions in the LIRE dataset are of western provenience. From these, 96,997 inscriptions are associated with the previously mentioned 889 western cities and 91,757 inscriptions are associated with the 360 cities for which we also have at least one occupation term (extracted from the inscriptions). Thus, the subsequent analysis relies on 91,757 inscriptions, representing approximately 67% of the LIRE dataset. These 67% of inscriptions contain 4,267 occupation terms, representing approximately 82% of the total number of occupation terms extracted across east and west (*N* = 5,222).

As explained in the Methods section, specialization measures the extent to which occupations associated with a city are accumulated within one particular occupational sector. In our case, we use the sector division of individual occupations inspired by Harris [32]. Table 1 lists 10 cities with the highest specialization score according to *ZIw*. First, we can notice that the *ZIw* specialization score does not seem to be very much dependent on other important city attributes, namely population size, number of inscriptions and number of occupations: among the 10 cities with the highest *ZIw* score there is both Rome, representing the largest city in the dataset, associated with the highest amount of inscriptions and occupational data as well, and two small towns, Petuaria (United Kingdom) and Casinum (Italy), with the estimate of 1,000 inhabitants and comparatively much smaller numbers of inscriptions and occupational terms.

**Table 1:** 10 cities with the highest *ZIw* score.

city	country	pop. est.	N inscriptions	N occupations	N occ. in largest sector	N sectors	ZIw	largest sector
Tibur	Italy	6767	235	52	31	10	2,36	Managerial
Alba Fucentia	Italy	4471	81	11	10	2	2,18	Managerial
Tusculum	Italy	2138	118	18	13	5	2,09	Managerial
Carmo	Spain	7172	8	8	8	1	2,08	Miscellaneous Services
Augusta Vindelicum	Germany	10608	200	10	9	2	2,07	Retail
Lavinium	Italy	3762	44	13	10	2	1,97	Managerial
Roma	Italy	923313	37765	1669	402	17	1,79	Managerial
Minturnae	Italy	4471	103	9	7	3	1,71	Managerial
Petuaria	United Kingdom	1000	7	5	5	1	1,61	Metal-Working
Casinum	Italy	1000	100	16	9	6	1,56	Managerial

The most specialized city is Tibur (modern Tivoli, Italy) located about 30 kilometers northeast of Rome, with 52 occupational terms dispersed across the 235 inscriptions in the LIRE dataset. The high extent of specialization of the city is driven by the fact that 31 of the occupations are from the Managerial sector. Upon a closer inspection of the data, we see that the most common occupation in Tibur is *curator*, the most common occupation overall, sometimes with individuals having multiple *curator* titles on one inscription (see S5 Data): e.g., senator Gaius Caesonius Macer Rufinianus (ca. 155–237 CE; HD030907) who held the title *curator rei publicae*, *c*. *aquarum et Miniciae*, and *c*. *alvei*, being responsible for finances, grain supply and water management in Rome [67]).

An interesting case is Carmo (modern Carmona, Spain) located in the fertile plain of the Guadalquivir River. The high specialization rank of this small city is caused by eight identified instances of *agrimensor* (a surveyor) on one inscription (EDCS-22400457) (see S6 Data). The reference is not to individuals but to collegia of land surveyors, with the collegia pointing to small settlements around Carmo. The date of the inscription (between 69 and 117 CE) coincides with the archeologically documented “explosion of rural settlement” connected to Flavian policy of establishing new municipia in Spain [68]. The environmental setting as well as the historical context both support Carmo’s specialization in agricultural production and the increased need for land survey specialists between 69 and 117 CE.

Petuaria (today Brough-on-Humber in Yorkshire, UK) is with 1,000 estimated inhabitants the smallest settlement among the most specialized ten. With only seven dated inscriptions, five contain a reference to an *argentarius*, assigning the Metal-Working specialization label to this coastal Roman military camp with an associated settlement. Archeologists have discovered five inscribed lead ingots at Petuaria, each weighing around 90 kg, and proposed a connection between the Yorkshire and Derbyshire ore-fields and the export of the processed ore via the Petuaria harbor [69].

A number of cities (Tibur, Tusculum, Alba Fucentia, Lavinium) on the top-ten list are old Italian cities and some of the earliest colonies of the Roman Republic. Located within a 30 to 60 km radius of the metropolis on the slopes of the Apennines, the spatial proximity to the largest market in the Mediterranean likely fostered their functional specialization which is dominated by curatorial services and the timber industry (*tignuarius* and *dendrophoros*) [61].

Turning to diversity, Table 2 lists the 10 cities with the highest *DI* score. Again, we encounter Rome among the top ten cities. It should not surprise us, since specialization and diversity are not exact opposites–a highly specialized city can maintain a substantial level of diversity as it has a broad base of crafts and industries [9]. However, in this regard, the size of the occupational data matters. For instance, a city with five occupations in total can be either highly specialized, with all five occupations accumulated in one sector only (see Petuaria on Table 1) or highly diversified, with the five occupations dispersed across as many sectors as possible, i.e. five. On the other hand, a city with a large number of occupation terms can reveal both high specialization and diversity. This explains the prominent position of Rome according to both measures.

**Table 2:** 10 cities with the highest *DI* score.

city	country	pop. est.	N inscriptions	N occupations	N occ. in largest sector	N sectors	DI
Capua	Italy	44416	861	46	8	13	9,45
Corduba	Spain	19404	488	30	5	10	8,49
Pompeii	Italy	9938	3228	55	9	12	8,47
Mogontiacum	Germany	19930	2924	48	10	13	8,29
Ostia	Italy	35016	2328	245	45	14	8,11
Pisae	Italy	3937	40	13	3	10	8,05
Puteoli	Italy	25091	1343	77	15	15	8,04
Roma	Italy	923313	37765	1669	402	17	7,73
Brigetio	Hungary	7999	378	14	3	9	7,54
Carnuntum (1)	Austria	7790	1103	19	4	10	7,37

The city with the highest *DI* score is Capua (modern Capua, Italy), located in the fertile Campanian plains and connected with Rome through *Via Appia*, with 46 occupations dispersed over 13 industry sectors. The high score of this metric indicates not only that there is a substantial number of sectors covered, but also that the sectors tend to be relatively equally distributed in this city when compared with other cities with a similar number of occupations and sectors. In Capua, there are seven sectors with at least four occupations (see S7 Data). Capua was known for its agricultural products, but also for its manufacturing of bronzes and production of luxurious items, such as perfumes (represented by three instances of *unguentarius*), or clothing [70].

Pisae (modern Pisa, Italy) located at the mouth of the Arno river is the city with the smallest population among the top ten *DI* score cities. Despite its small size, Pisae was an important trading center with vibrant sea-born commerce evidenced by the remains of 31 ships with cargoes of fruit, olives, wine and sand, from the second century BC to the seventh century CE [71, 72]. The inscriptions confirm existence of *collegium fabrum navalium Pisanorum*, a professional organization for ship builders, as well as evidence for timber industry located in Pisa (*fabri tignarii Pisani*) in the second century CE (EDCS-20402907) and numerous other professionals such as merchants, winemakers, goldsmiths, and dramatists (see S8 Data).

As an alternative to *DI*, we have also implemented *DIw*, which attempts to be more sensitive to the divergent sizes of occupational data (see Methods, Specialization and Diversity). But we have not been satisfied by its outcomes, as it seems to assign too high values to cities with small numbers of epigraphic data. We report the 10 cities with the highest *DIw* score as S9 Data.

### Population estimates, inscriptions and occupations

For the dataset of Western cities, we have evaluated statistical relationships between various combinations of variables. Taking into consideration its exceptional size, the city of Rome was excluded from these analyses as an outlier. S6 Fig offers an overview of relationships between numerous pairs of variables in the form of a correlation matrix. First of all, we took under consideration the fact that the number of identified occupations associated with each city is constrained and affected by the number of inscriptions located in the neighborhood of the city. Being aware of this constraint, we have implemented the *ZIw* score, weighing the original *ZI* score by the number of inscriptions. As a result, there is only a very weak statistical association between the number of inscriptions and the *ZIw* score (Pearson’s *r* = 0.11, *p* = 0.1). In the case of *RZIw* the association is even weaker (r = 0.04, p = 0.03). In other words, the relationship between our specialization measurements and the epigraphic data which have been used for their calculation is negligible.

The association between the number of inscriptions and the extent of diversification remains stronger. For obvious reasons, for a city with a higher number of inscriptions is comparably easier to have a high *DI* score than for a city with few extant inscriptions. Indeed, there is statistically a rather strong relationship between the number of inscriptions and the *DI* score (*r* = 0.52, *p*<0.001). The relationship is even stronger when the number of inscriptions is logarithmically normalized (*r* = 0.6, *p*<0.001). Technically, we can eliminate this relationship by employing the *DIw* score. But, as has been said before, we are not fully satisfied with its outcome, assigning high values to cities with very low numbers of inscriptions. Duranton and Puga [9] assert that larger cities will have higher diversity indices anyway. Thus, our results might also be interpreted as supporting this proposition. Despite this tendency, we should not ignore that among the 10 cities with the highest *DI* score there is still one small city (Pisae) and several other cities with less than 10,000 estimated inhabitants.

Further, we have made an attempt to explore the linkage between specialization or diversity of cities and their economic productivity or resilience. As a proxy for a city’s resilience, we calculated the ratio of inscriptions produced before and after 235 CE: a higher proportion of inscriptions produced after 235 CE is here interpreted as indicating higher economic resilience of a city in face of the third century crisis. Drawing on recent empirical studies of modern urban systems [10–12], we hypothesized that the cities with a higher sectoral diversity will reveal higher resilience than highly specialized cities.

However, in our data, we were not able to detect any statistical association between the two respective variables. These results do not necessarily mean that the link between sectoral diversity of cities and their resilience was missing in Roman antiquity, since the results can also be caused by inappropriate operationalization of the two factors or poor quality, insufficient representativeness of the data. Perhaps an assessment of economic prosperity versus resilience of ancient Roman cities might be possible via other archeological evidence, but that falls outside the scope of this paper.

### Division of labor and associations

We have also compared our results with results from [28]. The authors of [28] employ the same dataset of cities as our study and also rely on quantifying the epigraphic evidence to some extent. To operationalize the division of labor, they use the data on professional associations from [29], using the numbers of associations per city as a proxy for the overall diversity of socio-economic activities within the city. Since the association data from [29] are available only for 250 cities, the analysis of division of labor in [28] focuses exclusively on this subset of cities from [49]. From these 250 cities, 141 are among the 360 cities we use within our analysis of sectoral specialization and diversity. The comparison of our results with the results from [28] is therefore constrained to this subset of 141 cities and also excludes Rome.

First of all, our comparison reveals a relatively strong correlation between the number of associations listed in [29] and used by [28] and the raw number of occupations we extracted from the inscriptions (Pearson’s *r* = 0.69, *p*<0.001, see S10 Data). There is also a robust statistical relationship between the number of associations and the *DI* variable (*r* = 0.41, *p*<0.001). Thus, it is evident that the results of the two studies are compatible, at least to some extent. However, the authors of [28] further introduce another, more elaborated measure of functional diversity, D(N), which attempts to control for both unequal epigraphic sample sizes associated with each settlement and the effect of unequal population sizes. D(N) is calculated as a ratio of the number of distinct associations and the number of inscriptions multiplied by the population size. Measured this way, the statistical association between functional diversity of cities from [28] and the variables we use in our study is weak.

## Discussion

Undertaking quantitative analysis with historical datasets poses methodological and interpretive challenges. We rely on data extracted from a subset of surviving monuments, whose content maps poorly to modern terminology and is far from an unbiased and statistically sound sample. We interpret the results using modern urban economic theory under the assumption that it applies to ancient urban environments [27, 32, 33].

To extract the occupational data, we use a computer-assisted but to some extent manual method. Latin is a morphologically rich language. In such a case, many common computational text analysis methods require the language data to be morphologically preprocessed, ideally representing individual words in their dictionary-like (lemmatized) form, otherwise repeated occurrences of the same word cannot be detected. However, considering the fact that the language of inscriptions is highly formulaic, with missing sentence division, and full of alternative spellings and inconsistent word-order in compounds, the standard machine-learning models for lemmatization (pre-trained on Latin literary texts) do not perform well. The combination of a manual approach with rule-based computer processing gives us more control over the detection of desirable occupation terms, but the results are still far from being perfect, prone both to false-positives and false-negatives. While our coding sheets usually included several spelling variants of an occupation, we might have missed variations that we had not thought of. Our spot checks revealed minor issues where the occupation spelling did not fit our expectations and at least one occupation (out of 5,222) was misinterpreted. Over repeated iterations of the extraction process followed by a manual check of a sample of data we increased the number of detected occupations by 30%.

It is likely that we did not detect all occupational terms in the 136,190 qualifying inscriptions. Out of the 882 unique occupations in our list, only 387 were detected in the LIRE dataset. The remaining 495 occupations are missing because they either do not occur in the LIRE dataset (originating instead from papyri or other sources), occur there in a different form, or occur in inscriptions that lack temporal or spatial data, which were excluded from the source dataset. Our counts will therefore differ from studies that include undated inscriptions, such as Verboven’s list of 22 inscriptions with *negiotiatores* in Lugdunum [73]. We detected only three inscriptions with *negotiatores* in Lugdunum in our spatiotemporally-constrained dataset. While our filters are necessary in order to model the evolution of labor division in the Roman West through time, the study of urban sectoral specialization and diversification could be rerun on an unfiltered dataset including undated inscriptions.

Our measures of sectoral specialization and diversification in cities rest on several interpretive steps. Firstly, we identify unique professions on the basis of lists of occupations compiled by [29–31] and may undercount occupations. The assignment of occupation to an industry sector, furthermore, relies on a professional judgment and manual categorization of each term. Some occupations evaded categorization, such as *faber* (a worker), covering a range of occupations from a silversmith to shipbuilder. Among the 666 instances of *faber*, 566 had no modifier and were assigned to an Undefined category, while 100 had a specifying modifier (e.g. *faber argentarius*) and were categorized following Harris. Further, modern standards for industry sectors capture poorly the multifaceted nature of ancient occupations. The HISCO database, for example, separates production from trade, while ancient practitioners engaged in both simultaneously [61]. In such cases, we selected the production category over trade, potentially reducing the number of secondary traders.

Exploring labor division through time, a question arises. Does the increase in the number of unique occupations per 1,000 words mean an actual change, ie. a more differentiated labor force and an increasing number of professions, or is it merely a rhetorical change and a delayed manifestation of a fairly static labor force situation in the epigraphic medium? One could explain the temporal trend of growing frequency of occupations per 1,000 words as marking an ideological shift and a culture change as Roman society begins to recognize and value the societal contribution of tradesmen and professionals over the previous ideology that valued inherited status.

The first consideration of urban specialization and diversity concerns source data. Our results are based on a series of inferences and proxy data ranging from archeology-derived population estimates to fragmentary propaganda texts rather than systematic samples or Bureau of Labor census data. Our assessment of sectoral specialization and diversity thus hinges on coordinated actions and personal incentives of a series of individuals rather than directly reflecting actual functional diversity of entire ancient urban communities. We measure the reality that has percolated into epigraphic evidence, survived, was documented, digitized, and passed our quality checks.

The second consideration is also a caveat. *DI* and *ZIw* results suffer from an obvious shortcoming: collation in time which can have the following consequence. A city that was highly specialized at any given time but whose dominant sector shifted over time (e.g. from mining ores to producing and trading cloth) would through collation of occupations across multiple sectors appear as diversified. Our approach thus potentially inflates sectoral diversity and reduces sectoral specialization. Under ideal circumstances, we would have enough inscriptions to divide them into smaller 25-year timeblocks and generate indices on the basis of contemporaries. While we can divide the dataset, the resulting numbers will be so low as to be meaningless. Mindful of the risk of generating more diversity than there actually was, we can rest assured that the index of specialization is solid.

Looking at the top-ten lists in both *DI* and *ZIw* measures, both contain reasonably well-excavated cities with large collections of epigraphy (Ostia, Pompei, Rome, Carnuntum), continually occupied sites (Padova, Mainz, Cordoba) or old Italian cities whose long use span holds the promise of well established “epigraphic habit”. The *ZIw* list, however, shows relatively low two-digit numbers of occupations per city with the exception of Rome and Tibur. The *DI* list has high two-digit occupation numbers, but again, only Rome and Ostia are in the three and four-digit range. Many cities in the *DI* list are harbors, which fits the expectation that multiple sectors would coexist in a central node in a transport network. Cities in the specialized list have more idiosyncratic geographic settings, but show some historical commonality with Italian cities and Managerial sector dominating, a situation that is hardly unexpected in the heartland of the Empire. The cities in each list nonetheless need to be taken with a grain of salt, as the custom of committing one’s achievements to stone and the preservation rate of epigraphic monuments may have varied considerably from one urban context to another. The indices of specialization and diversification remain mere heuristical proxies, highly dependent on the quality of the underlying data. As such, instead of employing them to conduct large-scale comparative studies, they might be more useful as starting points for archeological and historical investigation of economic growth and decline in the respective cities during the third and fourth centuries CE.

Inscriptions are traditionally considered an urban phenomenon. On the example of Latin inscriptions in the LIRE dataset that contain both date and spatial information, we have confirmed this to be true only partially. The large cities with the largest size of occupations, such as Rome or Ostia, confirm an early onset of publication of inscriptions, while mid-sized and small cities lag behind. Against all expectations, we have discovered that the famous Severan peak (193–235 CE) in the production of Latin inscriptions was driven mostly by small cities and settlements in the rural area. Occupations follow similar patterns, appearing first in larger cities at the time of Imperial building programs, while in the late second century most occupational evidence comes from smaller settlements and even rural areas. Whether that might be caused by increasing urbanism within the Empire or cultural norms spread by the merchants or army deserves further investigation.

Taking a comprehensive dataset of Latin inscriptions and a fully transparent and scripted set of methods, we were able to explore ancient urban dynamics through the prism of modern urban economics. We mapped the occupational data extracted from epigraphic evidence onto urban contexts differentiated by population size and classified them by labor sectors in order to assess the sector ratios and the dependence of different professions on specific urban contexts. The results correspond to our expectations for tertiary sector expansion in larger cities. We then zoomed further to the level of individual cities, constructing employment profiles from all recovered inscriptions and translated these into weighted measures of sectoral diversity and specialization. However, the results of our measurements evade easy interpretation. While cities with high diversity index show some shared patterns such as being trading hubs or harbors, cities with high sectoral specialization require case by case review of geographic setting, epigraphic and archaeological evidence to interpret which sector dominates and why. Our analyses concerning the impact of these indices on productivity and resilience of cities remained inconclusive. It becomes obvious that our measures need to be taken as a rough guide and with a grain of salt as their reliability and representativeness suffer from the collation of highly fragmentary evidence. Thus, it appears that an appropriate interpretation of the results of our quantitative analyses requires a continuous dialogue between the “distant reading” and “close reading” approach to the sources.

## Supporting information

S1 Data Durations table. (CSV)

S2 Data Occupations list. (CSV)

S3 Data Occupations counts. (CSV)

S4 Data Occupations by context—descriptive statistics. (CSV)

S5 Data Occupational inscriptions–Tibur. (CSV)

S6 Data Occupational inscriptions–Carmo. (CSV)

S7 Data Occupational inscriptions–Capua. (CSV)

S8 Data Occupational inscriptions–Pisae. (CSV)

S9 Data 10 cities with the highest *DIw* diversity score. (CSV)

S10 Data Correlation matrix—comparison. (CSV)

S1 Fig Durations histogram. (TIF)

S2 Fig Occupations by inscription type. (TIF)

S3 Fig Occupations across urban contexts (Rome separately). (TIF)

S4 Fig Occupations by industry type across urban contexts (Rome separately). (TIF)

S5 Fig Occupations by industry type across urban contexts (HISCO). (TIF)

S6 Fig Correlation matrix. (TIF)

S1 File (TXT)
