# Supplementary figures and images for: Division of labor, specialization and diversity in the ancient Roman cities: A quantitative approach to Latin epigraphy
Source: PLoS One. 2022 Jun 16;17(6):e0269869. doi: 10.1371/journal.pone.0269869 (PMC9202948; doi:10.1371/journal.pone.0269869)

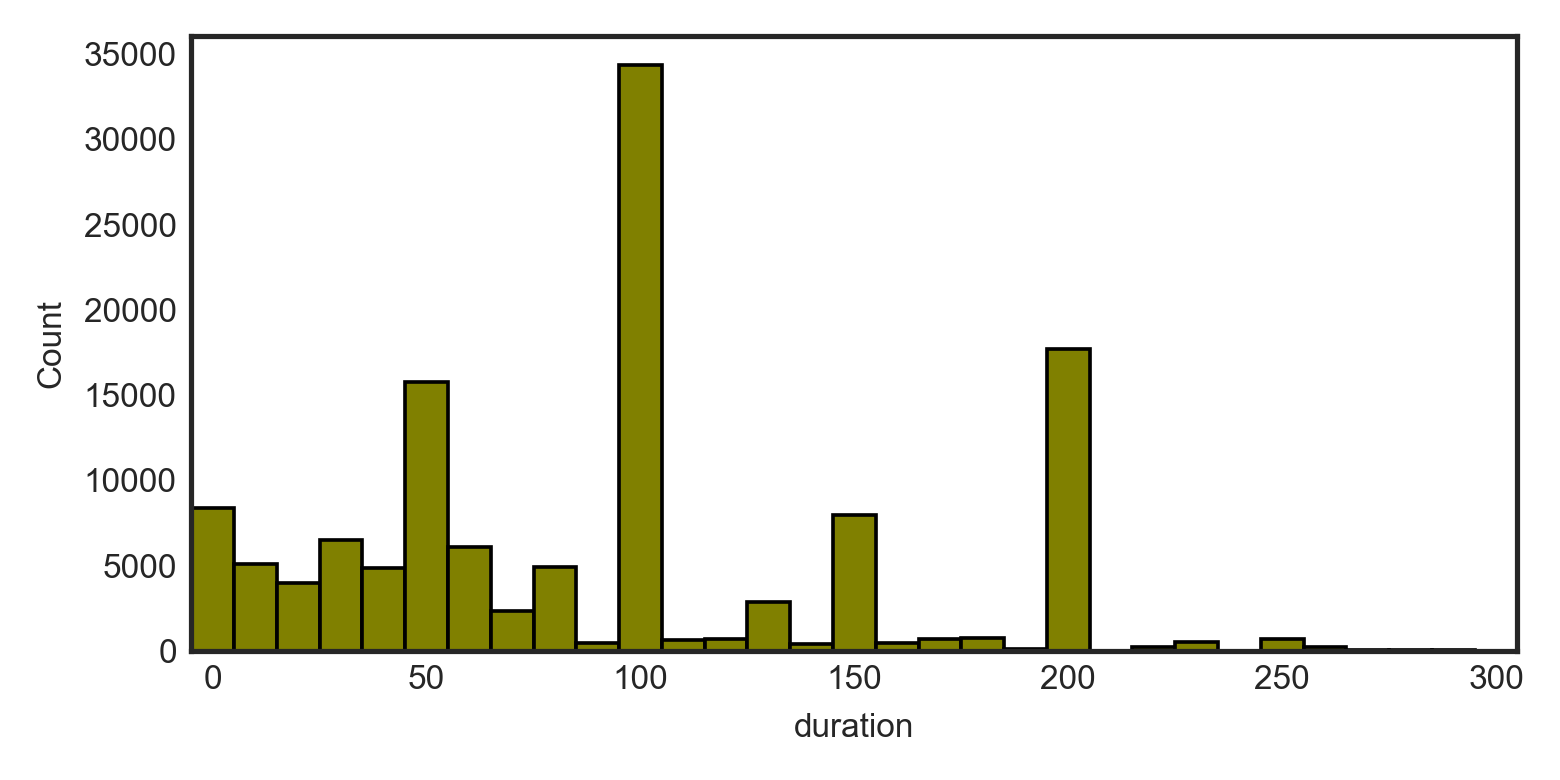

Supplement: S1 Fig — (TIF) [file pone.0269869.s011.tif]

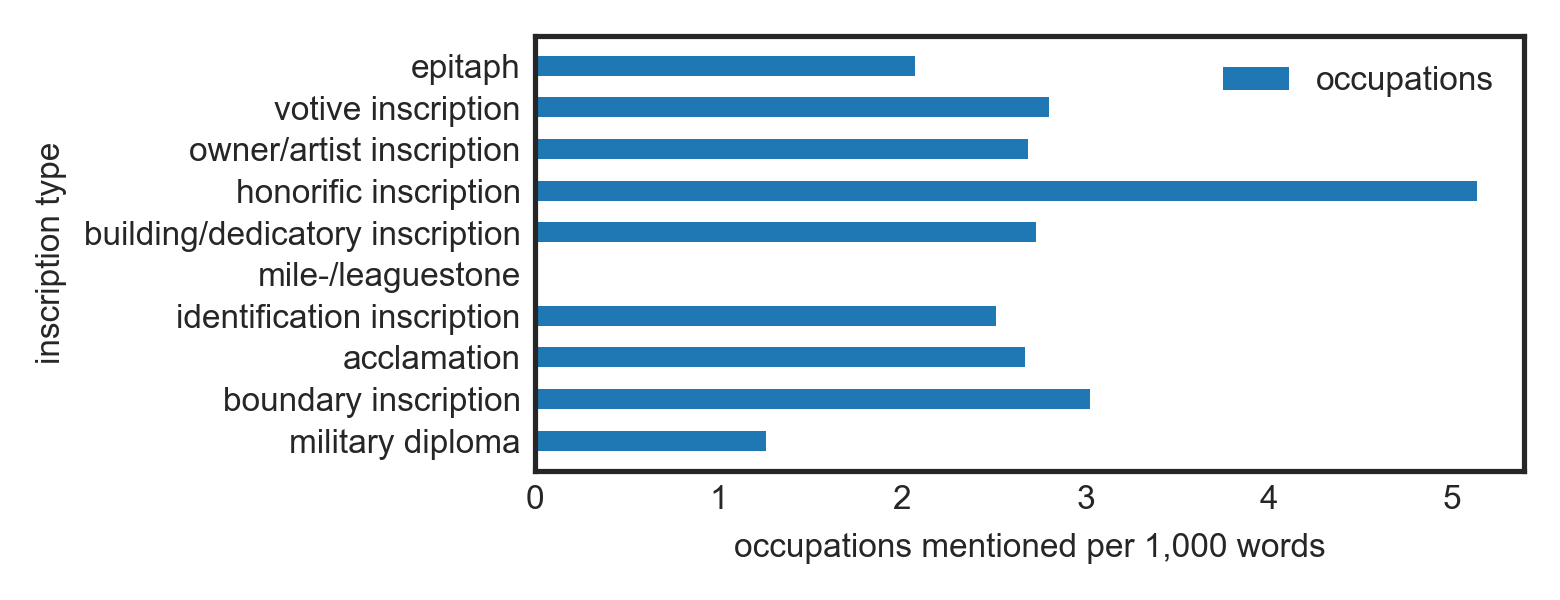

Supplement: S2 Fig — (TIF) [file pone.0269869.s012.tif]

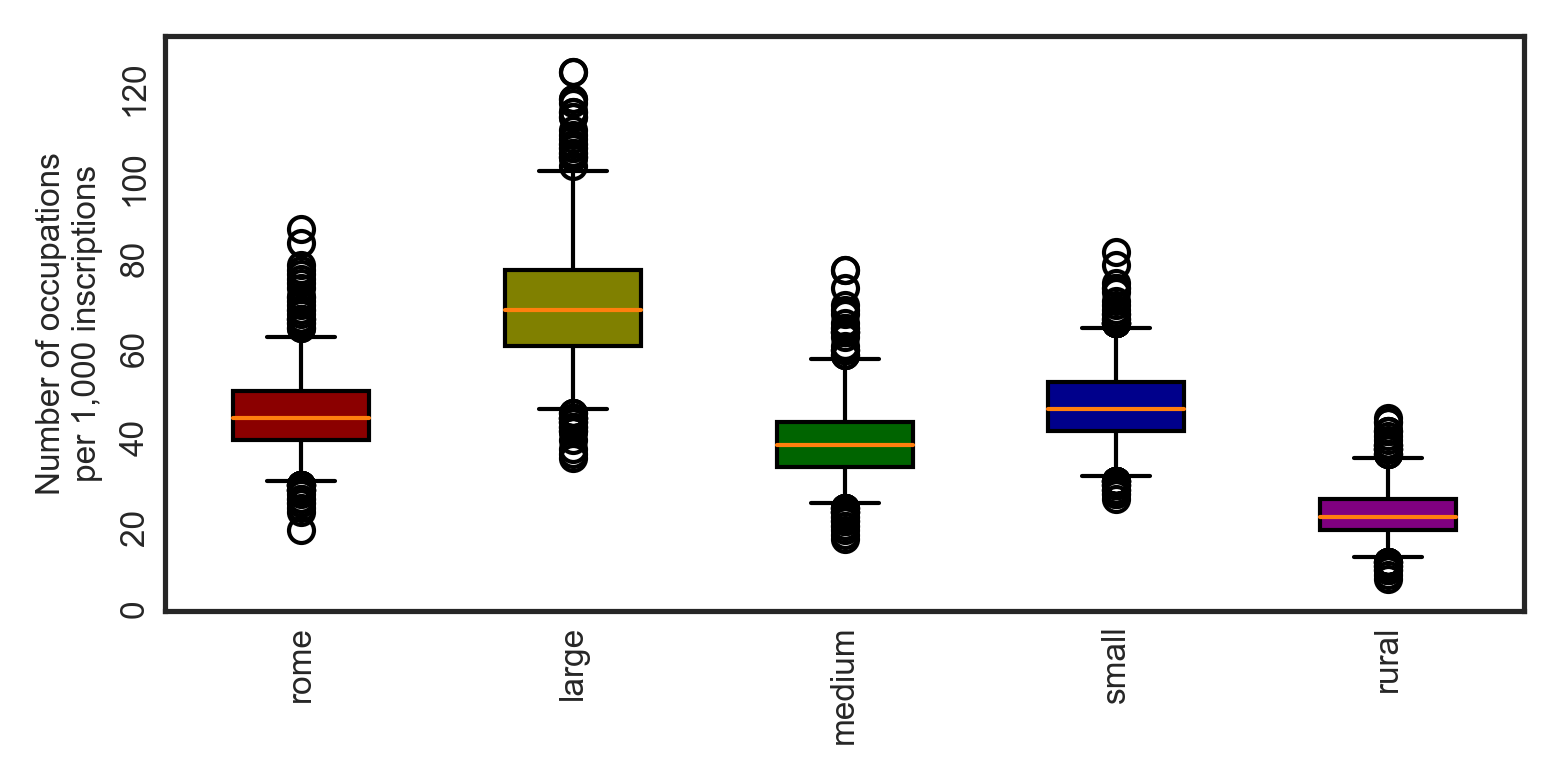

Supplement: S3 Fig — (TIF) [file pone.0269869.s013.tif]

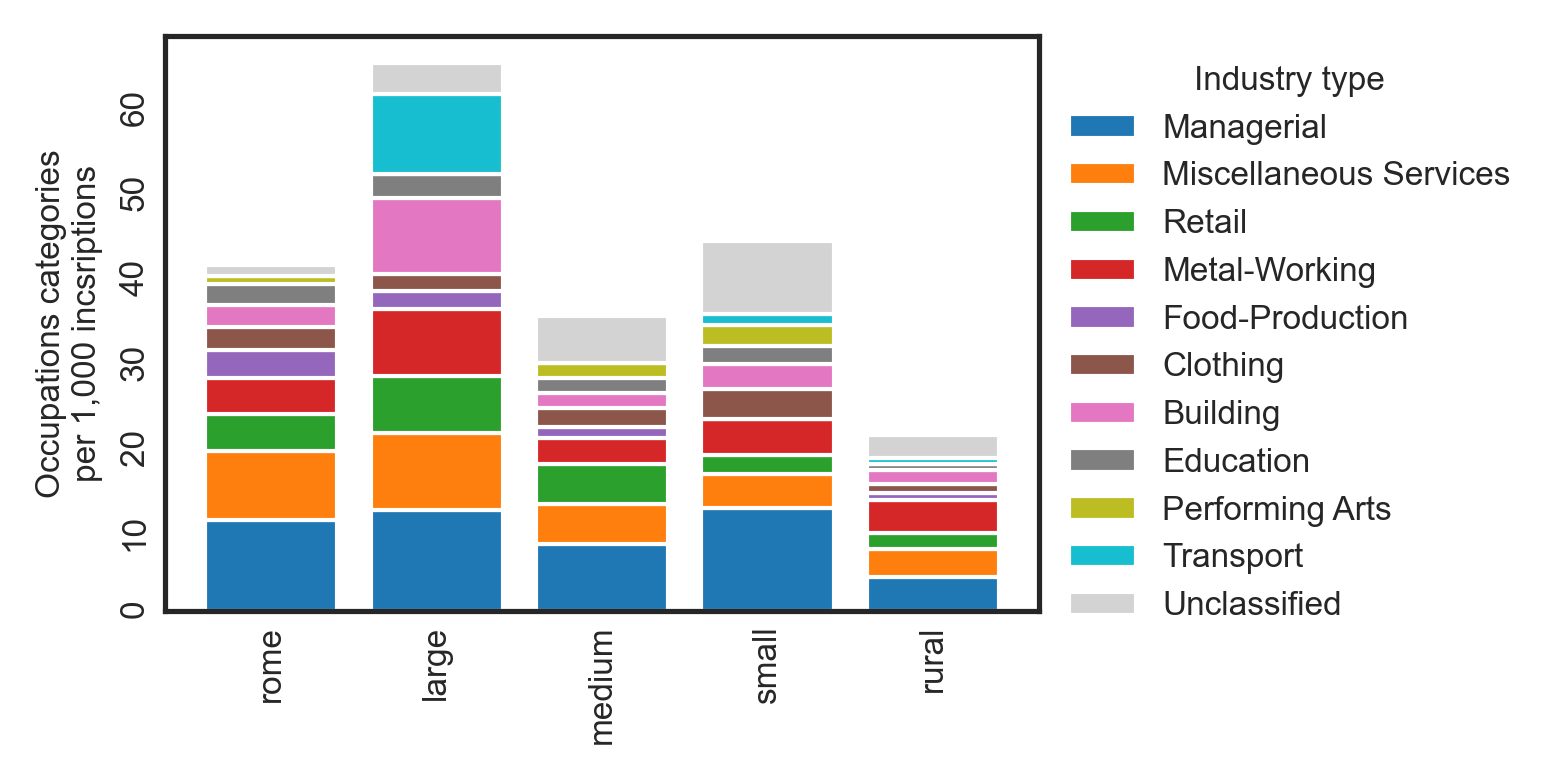

Supplement: S4 Fig — (TIF) [file pone.0269869.s014.tif]

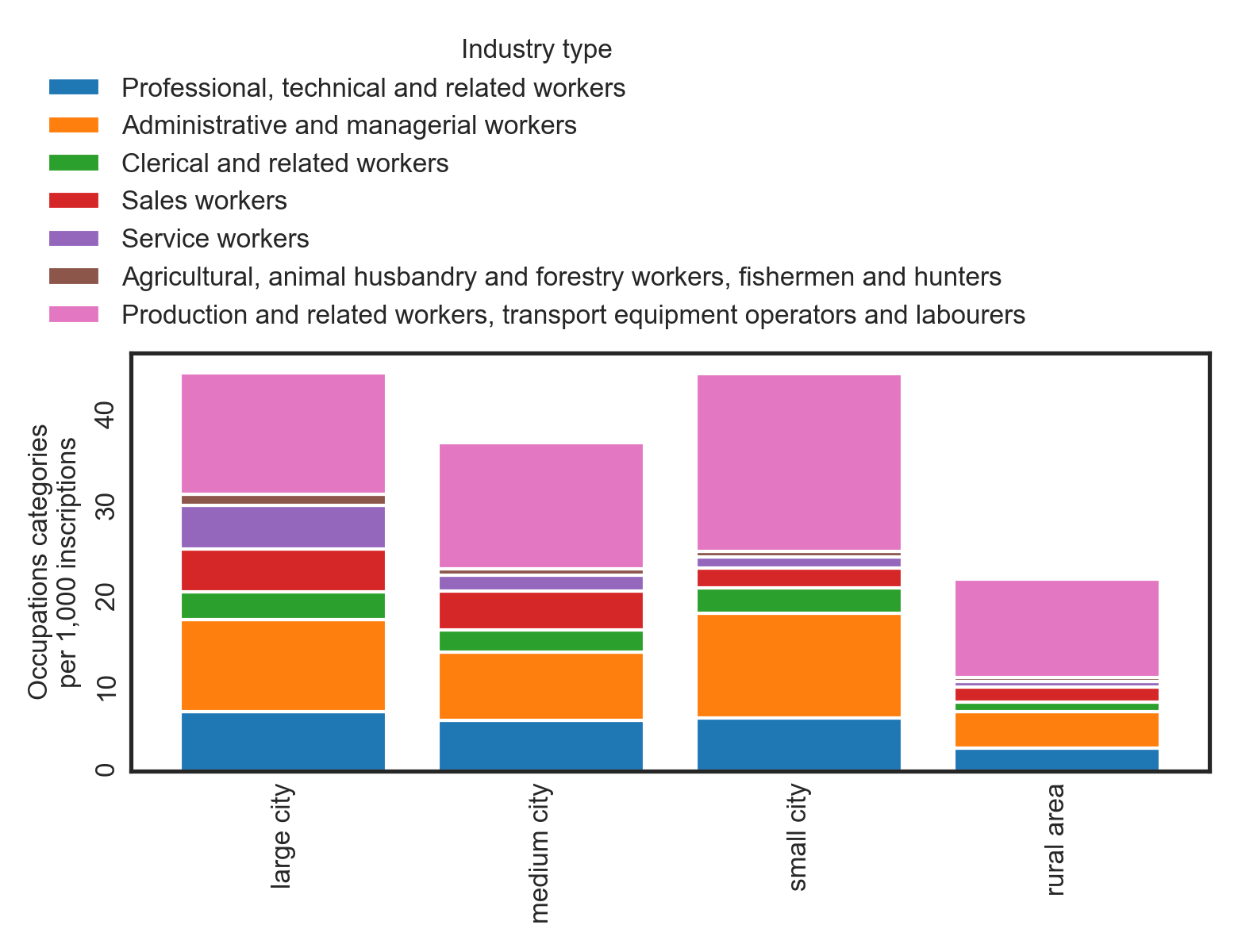

Supplement: S5 Fig — (TIF) [file pone.0269869.s015.tif]

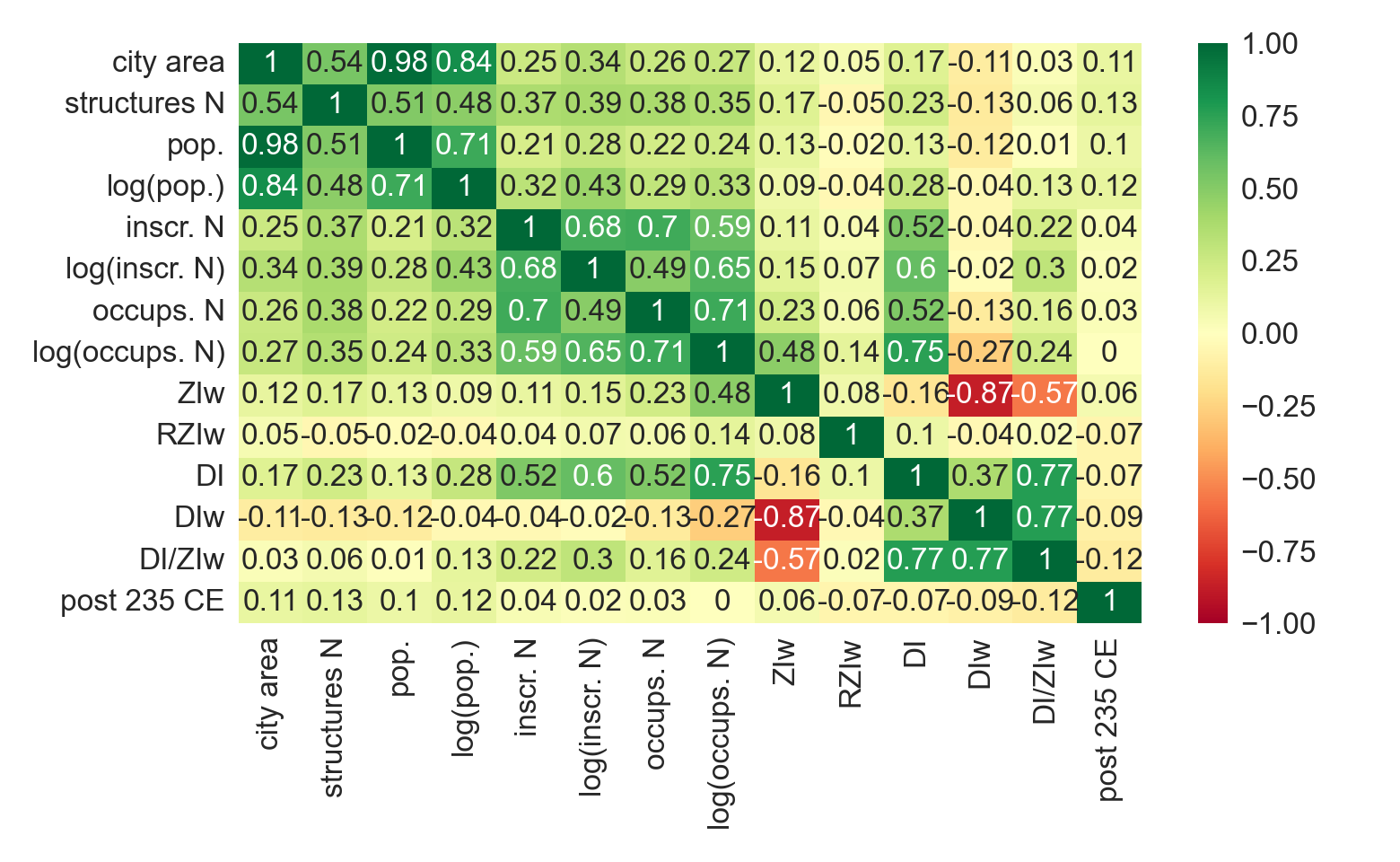

Supplement: S6 Fig — (TIF) [file pone.0269869.s016.tif]
